# Supplementary material for: Predicting visual function by interpreting a neuronal wiring diagram
Source: Nature. 2024 Oct 2;634(8032):113–23. doi: 10.1038/s41586-024-07953-5 (PMC11446822; doi:10.1038/s41586-024-07953-5)
Supplement: Supplementary file 6 — CRF and ERF predictions for individual TmY4 and TmY9 cells. Analogous to Supplementary Data 3, but for TmY target types. Shown are the top four monosynaptic pathways, the strongest pathway passing through each of the top ten intermediary types (ranking from Extended Data Fig. 7), and the trisynaptic pathway Tm1–TmY–Dm3–TmY (see the section entitled Prediction of spatial normalization). [file 41586_2024_7953_MOESM6_ESM.zip › DataS4/TmY9qΓèÑ/720575940608607324.pdf]

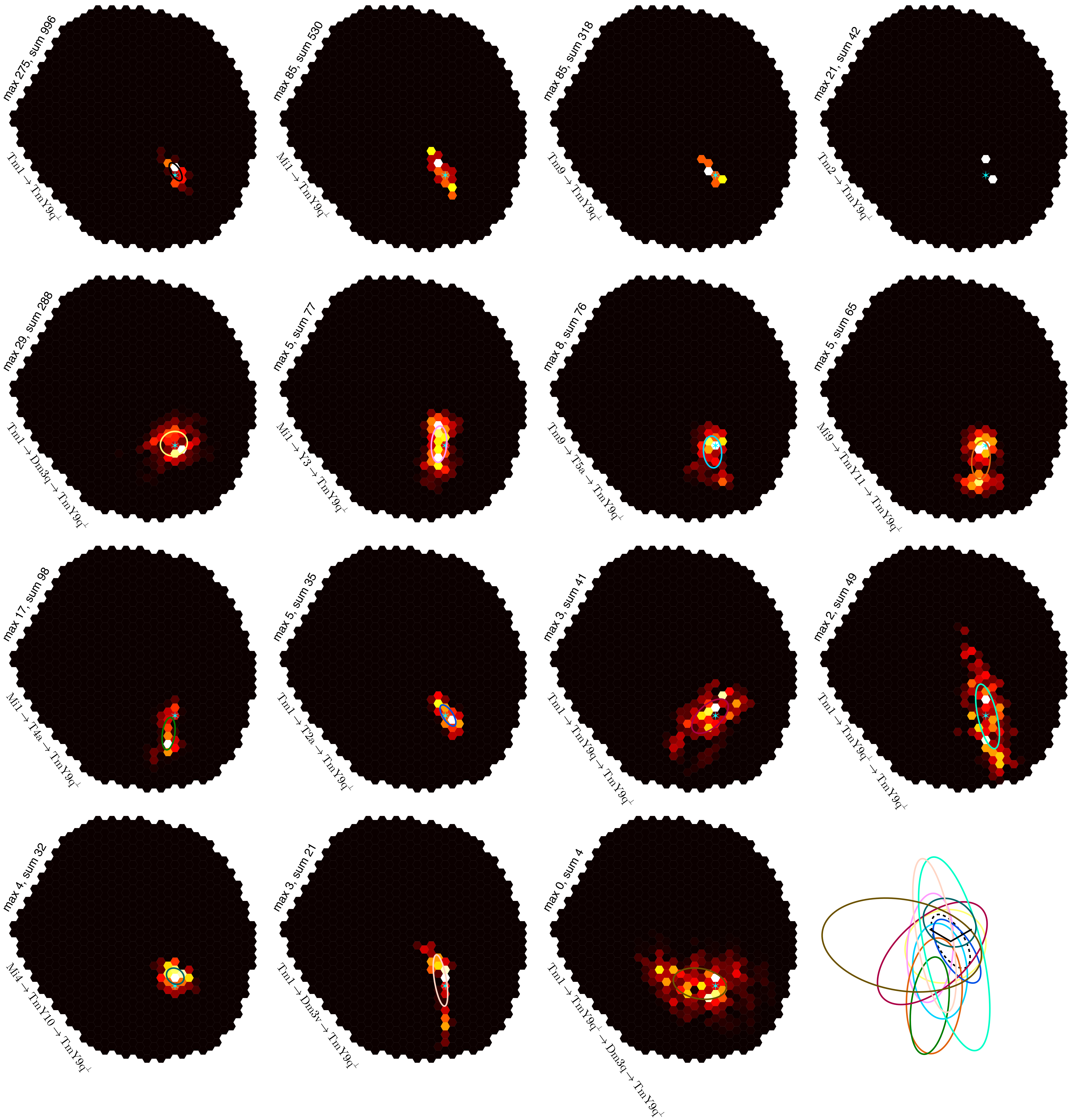

max 275, sum 996
Tm1 → TmY9q⊥
max 85, sum 530
Mi1 → TmY9q⊥
max 85, sum 318
Tm9 → TmY9q⊥
max 21, sum 42
Tm2 → TmY9q⊥
max 29, sum 288
Tm1 → Dm3q → TmY9q⊥
max 5, sum 77
Mi1 → Y3 → TmY9q⊥
max 8, sum 76
Tm9 → T5a → TmY9q⊥
max 5, sum 65
Mi9 → TmY11 → TmY9q⊥
max 17, sum 98
Mi1 → T4a → TmY9q⊥
max 5, sum 35
Tm1 → T2a → TmY9q⊥
max 3, sum 41
Tm1 → TmY9q⊥ → TmY9q⊥
max 2, sum 49
Tm1 → TmY9q⊥ → TmY9q⊥
max 4, sum 32
Mi4 → TmY10 → TmY9q⊥
max 3, sum 21
Tm1 → Dm3v → TmY9q⊥
max 0, sum 4
Tm1 → TmY9q⊥ → Dm3q → TmY9q⊥
